# Uncommon Cause of Acute Drug-induced Liver Injury Following Mammoplasty

**DOI:** 10.4021/gr2010.06.210w

**Published:** 2010-07-20

**Authors:** Atul Singla, Hazem T. Hammad, Ghassan M. Hammoud

**Affiliations:** a Department of Internal Medicine, University of Missouri School of Medicine, One Hospital Drive, Columbia, MO 65212, USA; b Division of Gastroenterology, Department of Internal Medicine, University of Missouri School of Medicine, One Hospital Drive, Columbia, MO 65212, USA

**Keywords:** Cephalexin, Hepatitis, Drug-induced

## Abstract

Cephalexin is a well tolerated antimicrobial and hepatic injury is an infrequent occurrence with its use. We here describe a 21-year-old female who presented with jaundice and elevated liver enzymes after 4 weeks completion of 10 day course of cephalexin, prescribed prophylactically after mammoplasty. Extensive work up including all causes of hepatitis was within normal limits and she improved with conservative management. This case highlights the need to suspect drug induced liver injury in cases of jaundice and cephalexin use.

## Case Report

**Figure 1:**
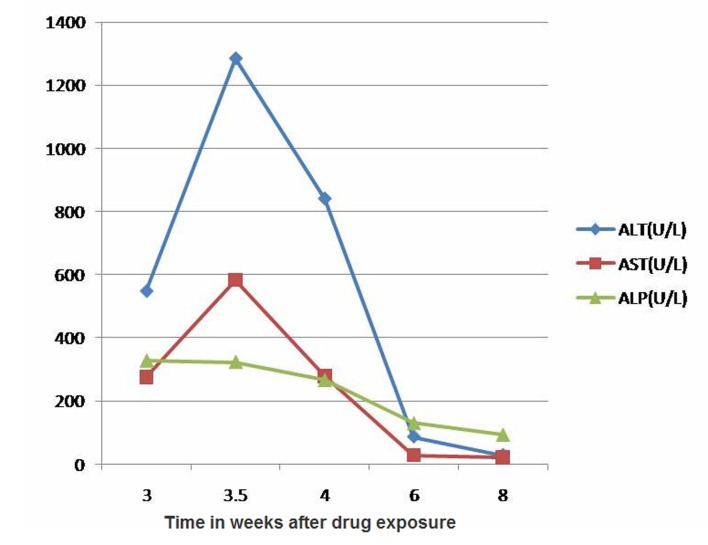
Changes of aminotransferases and alkaline phosphatase.

A 21-year-old previously healthy female with recent history of uneventful mammoplasty presented with one-week history of right upper quadrant abdominal pain, skin rash, muscle aches, jaundice, dark urine and pruritus one month after her surgery. She had received Cephalexin 500 mg capsule three times daily prophylactically for 10 days postoperatively. She had no history of fever, chills, weight loss, joint pains, travel abroad or ill contacts. She denied alcohol intake or exposure to any herbal or over the counter supplements. Her surgical records revealed no hypotensive episodes perioperatively. Physical examination revealed alert young woman with icteric sclera. She had mild tenderness in the right upper quadrant and had no hepatosplenomegaly or ascites. Skin examination revealed a macular rash over the right flank with no vesicles. Laboratory tests were significant for WBC 9300 cells per microliter with 7.2% eosinophils, alanine aminotransferase (ALT) 1280 U/L (normal range 10 - 60 U/L), aspartate aminotransferase (AST) 580 U/L (normal range 10 - 40 U/L), alkaline phosphatase (ALP) 323 U/L (normal range 38 - 126 U/L), gamma glutamyl transpeptidase (GGTP) 323 U/L (normal range 7 - 50 U/L), total bilirubin 4.3 mg/dL with a conjugated fraction of 2.9 mg/dL. Serum total protein, albumin, LDH, amylase, creatinine, TSH and prothrombin time were all normal. Serologic tests for acute viral hepatitis A, B and C, CMV, HSV, and EBV were negative. Her serum autoantibodies and tests for metabolic liver diseases such as hemochromatosis, Wilson disease and alpha-1 antitrypsin deficiency were negative. Ultrasound of the abdomen showed normal echogenicity of the liver. The gall bladder appeared contracted with no stones or evidence of acute cholecystitis. Doppler studies of the portal and hepatic veins were normal. With close follow-up, her abdominal pain and jaundice resolved. Cholestyramine alleviated her pruritus and she had a complete normalization of liver transaminases eight weeks after exposure to cephalexin (Fig. 1). Liver biopsy was not performed.

## Discussion

Cephalexin is a first generation cephalosporin and a well tolerated antibiotic which has been widely used for variety of skin and soft tissue infections. Gastrointestinal disturbances such as nausea, vomiting, diarrhea and abdominal pain are common. Mild elevations (less than 2 - 3 fold) in serum transaminases are common but severe hepatocellular injury is very rare. Other groups of cephalosporin have been implicated in drug-induced liver injury. Chen et al reported acute hepatitis with cefdinir [1]. Pacik reported two cases of prophylactic cephazolin induced hepatitis after augmentation Mammoplasty [2].

Search of medical literature in English language from January 1969 to January 2010 including Pubmed, MEDLINE, WHO database, Cochrane database, PDR, Food and drug administration (FDA) website reveals a single case report of cephalexin induced hepatic cholestasis. Skoog et al reported a 51-year-old male who received cephalexin for 10 days and developed dark colored urine and pale colored stools three days after taking cephalexin [3]. Within 24 hours of completing therapy, fever, hives and jaundice ensue. Liver biopsy showed panacinar hepatitis and extensive cholestasis. Our patient was prophylactically treated with cephalexin after mammoplasty and developed jaundice and marked elevation (more than 20 fold) of liver transaminases four weeks after exposure to cephalexin. Extensive history and laboratory tests revealed no risk factors for viral hepatitis or drug-drug interaction. Her liver enzyme elevation, the presence of eosinophilic leukocytosis and skin rash pointed towards hypersensitivity category of idiosyncratic reactions of severe hepatocellular injury. Moreover, the close temporal relationship between completion of cephalexin therapy and onset of symptoms with lack of other causes of hepatitis strongly suggested drug-induced liver injury.

In general, there are no standard criteria or biochemical tests for diagnosis of drug induced hepatotoxicity. Few models have been developed that attempt to correlate causality of drug toxicity into objective criteria such as the Naranjo Adverse Reactions Probability Scale (NADRPS), Councils for International Organizations of Medical Sciences (CIOMS)\Roussel-Uclaf Causality Assessment Method (RUCAM) scale, and the Maria & Victorino (M & V) clinical scale [4]. However, none of these models address all risk factors and neither is routinely used in clinical practice. In our patient, NADRPS indicated a ‘robable’ (score = 6) and RUCAM a ‘possible’ (score = 10) relationship between the drug and liver injury. Serum bilirubin more than 3 times the upper limit of normal in association with elevated aminotransferases, is associated with bad prognosis (Hy’s Law) [5]. Fortunately, our patient had a benign course with complete recovery. In conclusion, cephalexin is a well tolerated antimicrobial agent and to our knowledge, severe hepatocellular injury is exceedingly rare if ever reported. Clinicians should be aware of the potential severe hepatotoxicity associated with cephalexin.
